# CBX3 promotes colon cancer cell proliferation by CDK6 kinase-independent function during cell cycle

**DOI:** 10.18632/oncotarget.15253

**Published:** 2017-02-10

**Authors:** Yao Fan, Haiping Li, Xiaolong Liang, Zheng Xiang

**Affiliations:** ^1^ Chongqing Key Laboratory of Department of General Surgery, Chongqing Medical University, Chongqing, China; ^2^ Department of Gastrointestinal Surgery, The First Affiliated Hospital of Chongqing Medical University, Chongqing, China

**Keywords:** colon cancer, CBX3, CDK6, p21, proliferation

## Abstract

Heterochromatin protein 1γ (CBX3) links histone methylation marks to transcriptional silence, DNA repair and RNA splicing, but a role for CBX3 in cancer remains largely unknown. In this study, we show that CBX3 in colon cancer cells promotes the progression of the cell cycle and proliferation *in vitro* and *in vivo*. Cell cycle (G1 phase to S phase) related gene CDK6 and p21 were further identified as targets of CBX3. In addition, we found that enhancing CDK6 suppresses cell proliferation by upregulating inhibitor p21 in the absence of CBX3, and this function is independent of the kinase activity of CDK6. Our results demonstrate a key role of CBX3 in colon carcinogenesis via suppressing the expression of CDK6/p21, which may disrupt the role of CDK6 in transcriptionally regulating p21, as part of a negative feedback loop to limit CDK6 excessive activation.

## INTRODUCTION

The histone proteins as well as several other proteins are essential elements of heterochromatin formation, and are associated with gene transcriptional repression or silencing [1]. The mammalian heterochromatin protein 1 is a highly conserved non-histone chromosomal protein and a major component of heterochromatin. Its orthologs include CBX5, CBX3 and CBX1, which are concentrated at pericentric heterochromatin or euchromatic sites, each containing a chromodomain [2]. Methylation of histone H3 lysine 9 (H3K9) can be recognized by the CBX3 chromodomain [3]. Dynamic CBX3 binding to H3K9 is dependent on the histone methyltransferase Suv339H1 and maintains stable heterochromatin domains to regulate gene expression [4]. In addition, CBX3 can recruit various cofactors which perform functions in intracellular biological processes by binding methylated H3K9. These functions include RNA alternative splicing, DNA damage response, transcription elongation, cell growth and differentiation [5–10]. Recently, although gene expression assays have revealed that CBX3 is linked with lung cancer, osteosarcoma, gastric cardia adenocarcinoma and colorectal cancer, among other diseases [11–15], the specific mechanisms by which CBX3 promotes cancer progression are poorly understood.

Cyclin dependent kinases (CDKs) are core parts of cell cycle regulation, containing over 20 members [16]. CDKs in binding and catalyzing cyclins are necessary to drive progression of the cell cycle. CDK4/CDK6-cyclinD complexes enable the cells to pass from G1 to S phase, but these proteins are critically required for proliferation only in selected cells types [17, 18]. Experiments with gene-deficient mice for CDK4 or CDK6 are viable [19], then lacking CDK4 and CDK6 die of severe anemia and hematopoietic abnormalities [20]. Partial experiments demonstrate D-cyclins enhancing interaction with CDK2 or cyclinD-CDK4/CDK6-independent mechanism. However, several are also involved in transcriptional regulation, DNA damage repair, stem-cell self-renewal, metabolism, spermatogenesis and neuronal function [21]. Recent researches focus on the role of CDK6 in transcriptional regulation. CDK6 acts as a cofactor targeting gene promoter, including IL-1, IL-8, EGRI [22–25]. The most prominent report that a kinase-independent function of CDK6 links cell cycle to tumor angiogenesis shows CDK6 participating in p16 and VEGF-A transcriptional regulation in lymphoid malignancies [26], CDK6 acts as a transcriptional regulator on the p16INK4a promoter providing an internal safeguard by negative feedback loop. However, CDK6 kinase-independent function in colorectal cancer has never been reported.

To investigate the mechanism by which CBX3 promotes colon cancer, we deleted CBX3 in HCT116 with the CRISPR/Cas9 editing system and found that cell cycle progression was curbed in G1 phase and proliferation was inhibited. Gene expression analysis reveals that CDK6/p21 is upregulated. In addition, CDK6 is able to directly regulate p21 expression in a kinase-independent manner. Therefore, we identify a new mechanism by which CBX3 promotes colon cancer progression via the CDK6/p21 pathway during cell cycle.

## RESULTS

### CRISPR/Cas9 induced CBX3 genome mutation

**Figure 1:**
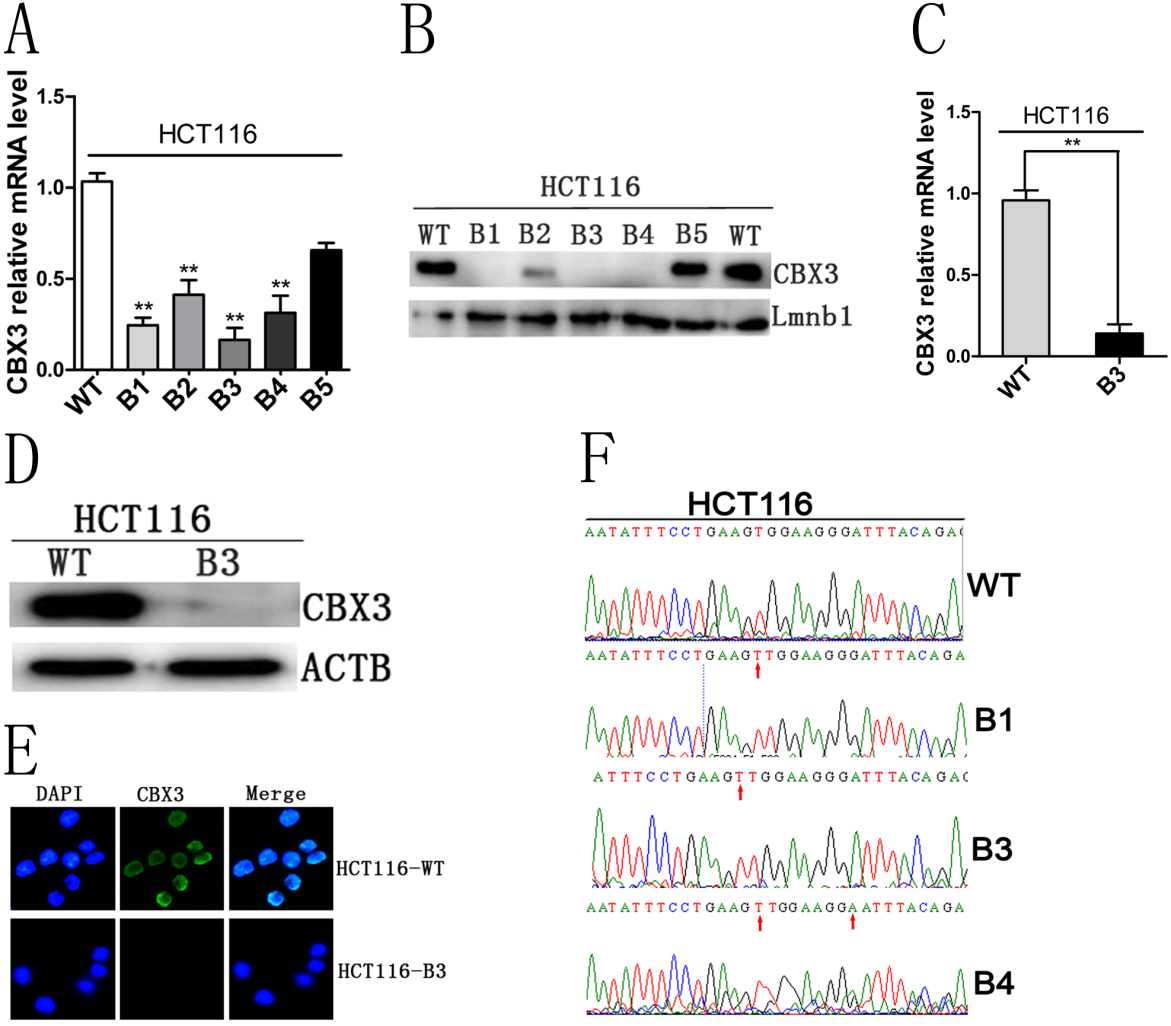
CRISPR/cas9 editing system induced the deletion of CBX3 protein **A**. Quantitative real-time PCR (qRT-PCR) analysed mRNA level of CBX3 in HCT116-B1, B2, B3, B4 and B5 colony cell lines. The mean±SD were shown from three independent experiment, **P<0.01 compared with control. **B**. Immunoblot for CBX3 of five colony cell lines derived from transfecting px330 plasmid in which Lmnb1 served as a loading control. **C**. qRT-PCR analysis mRNA level of CBX3 from HCT116-B3 cell lines continuously cultured for 5 generations. The mean±SD were shown from three independent experiments. **P<0.01 compared with the control. **D**. Immunoblot for CBX3 of HCT116-B3 and HCT116-WT cells, ACTB served as a loading control. **E**. Cell immunofluorescence was performed in HCT116-B3 and HCT116-WT cells. Nuclei were identified by DAPI, immunofluorescence staining for CBX3. Scale bar=10 um. **F**. Base T was inserted in NGG site in B1, B3 and B4, base G mutation is base A in B4, verified by sequencing the CBX3 genome.

To identify the relationship between CBX3 and the pathological process of colon cancer, the CRISPR/cas9 genome editing system was used to modulate the CBX3 gene in HCT116 cells. sgRNA was designed and Px330 plasmid was constructed as shown in (Supplementary Figure 1). Several colony cell lines were established by from transfected cells. We found that the expression of CBX3 in the cell line, HCT116-B3 was significantly lower than that in others (Figure 1A, 1B, 1C and 1D). Furthermore, no fluorescence was detected in HCT116-B3 cells by cell immunofluorescence when immunostained with anti-CBX3 fluorescence antibody (Figure 1E). To verify that the deletion of CBX3 was due to CRISPR/cas9 editing, CBX3 genome was sequenced in HCT116-WT, B1, B3 and B4 cells. We found that an additional base T was inserted in the NGG site to change the CBX3 genome sequence (Figure 1F).

### CBX3 promoted colon cancer cell cycle progression and proliferation *in vitro*

**Figure 2:**
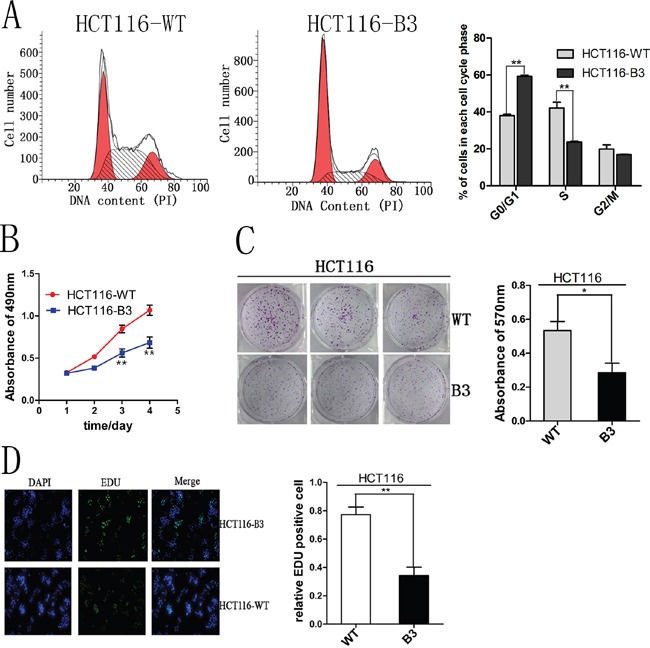
CBX3 deletion inhibited cell cycle progression and proliferation *in vitro* **A**. Flow cytometry assays were performed to analyze cell cycle in HCT116-WT and B3 cells. Values at different stages of cell cycle represent mean±SD from three independent experiments. **P<0.01 compared with control. **B**. Colon cancer cell proliferation were detected by MTS and absorbance at 490 nm at different time points is shown. Each data point represents mean±SD from three independent experiments. **P<0.01 compared with control. **C**. Colony formation assays were performed using HCT116-WT and HCT116-B3 cells. Colony number were analyzed by measuring absorbance at 570 nm, The results were shown as mean±SD from three independent experiments. *P<0.05 compared with control. **D**. The effect of CBX3 deletion on the growth of colon cancer HCT116 was analyzed by EdU proliferation assay. **P<0.01 compared with control.

To investigate the effect of the deletion of CBX3 on colon cancer cell growth, we used flow cytometry to analyze cell cycle and apoptosis. These results shown that cell cycle was significantly inhibited in G1 phase compared with the control (Figure 2A and Supplementary Figure 3A), but we did not find any change in apoptosis (Supplementary Figure 2A and 2B). Cell proliferation was also analyzed by MTS assay, and the deletion of CBX3 in colon cancer cells evidently reduced cell growth rate (Figure 2B and Supplementary Figure 3C). To directly observe cell growth, colony formation assays were performed. A specified number of cells were seeded into plates and the deletion of CBX3 in the colon cancer cell line significantly reduced the numbers of colonies per dish after incubation for 5 days (Figure 2C and Supplementary Figure 3B). These results were further confirmed by EdU staining which could detect nucleotide analogue incorporation into replicated DNA (Figure 2D). These data indicate that CBX3 promotes colon cancer cell proliferation by curbing cell cycle G1-S phase transition.

### The deletion of CBX3 directly enforces the expression of CDK6 and p21

**Figure 3:**
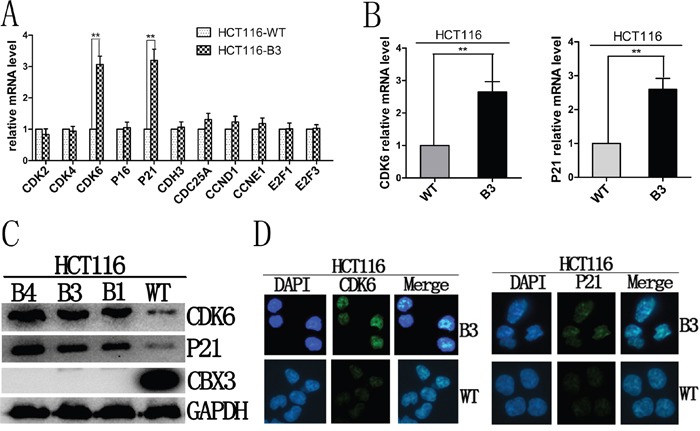
Deletion of CBX3 reduced the expression of CDK6 and p21 **A**. The mRNA abundances of CDK2, CDK4, CDK6, P16, P21, CDH3, CDC25A, CCND1, CCNE1, E2F1, E2F3 were analyzed with qRT-PCR in HCT116-B3 cells relative to the control. The results were shown as the mean±SD from three independent experiments. **P<0.01 compared with control. **B**. qRT-PCR were performed to analyze the mRNA level of CDK6 and p21 of HCT116-B3 relative to control. The results were shown as the mean±SD from three independent experiments. **P<0.01 compared with control. **C**. Immunoblot for CDK6, p21 and CBX3 derived from HCT116-WT, B1, B3 and B4 cells,, with GAPDH served as a loading control. **D**. Cell immunofluorescence were performed using HCT116-WT and B3 cells. Nuclei were identified by DAPI, immunofluorescence staining for CDK6 and p21. Scale bar=10 um.

To investigate the underlying mechanism, we examined the expression of several genes known to be important for cell cycle G1-S phase transition, including CDK2, CDK4, CDK6, p16, p21, CDH3, CDC25A, CCND1, CCNE1, E2F1 and E2F3 in HCT116-B3 and HCT116-WT cells (Figure 3A). We found the mRNA abundances of CDK6 and p21 consistently increased in the deletion of CBX3 cells (Figure 3B). Immunoblot results also revealed that the deletion of CBX3 causes high protein expression of CDK6 and p21 compared with control (Figure 3C). In order to determine whether elevated CDK6 and p21 in the cells in which CBX3 was deleted could be observed directly at the level of a single cell. Cell immunofluorescence staining for CDK6 and p21 showed a dramatic difference between HCT116-B3 and HCT116-WT (Figure 3D).

### CBX3 promotes xenograft tumor growth in correlation with CDK6 and p21 expression

**Figure 4:**
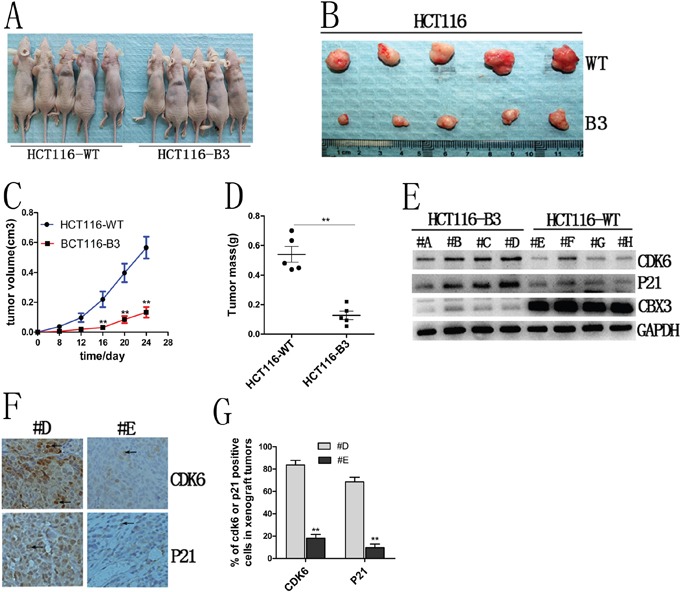
CBX3 deletion suppressed xenograft tumors growth in nude mice **A**. The growth of subcutaneous tumors in nude mice from HCT116-B3 or control cell growth (n=10). **B**. Tumor tissues derived from xenograft tumors in nude mice after 24 days. Top, tumor from control; bottom, tumor from the deletion of CBX3. **C**. The mean volume of xenograft tumors from HCT116-B3 versus WT, **P<0.01. **D**. The average tumor weight from HCT116-B3 versus WT, **P<0.01. **E**. xenograft tumors tissues protein were extracted from HCT116-B3 (n=4,#A,#B,#C,#D) and HCT116-WT (n=4,#E,#F,#G,#H), Immunoblot for CDK6, P21 and CBX3. GAPDH served as a loading control. **F**. Immunohistochemical stainings of xenograft tumor tissues from HCT116-B3 (#D) and control HCT116-WT (#E) for CDK6 and P21. Original magnification×400. **G**. Quantification of CDK6 or p21 positive cells from F picture, **P<0.01 compared with control.

To investigate the mechanism by which CBX3 directly promotes colon cancer cell proliferation *in vivo*, we performed xenograft tumor growth assays in nude mice and the size and weight of tumors were measured after 24 days. We found that there is a significant difference in tumor growth between HCT116-B3 and HCT116-WT (Figure 4A, 4B, 4C and 4D). These results suggest that CBX3 accelerates tumor formation *in vivo*.

We had identified that the deletion of CBX3 increases CDK6 and p21 expression in colon cancer cells. To further confirm this result, immunoblots were performed for CBX3, CDK6 and p21 in xenograft tumor tissue. We found that deletion of CBX3 significantly increased CDK6 and p21 protein abundances (Figure 4E). In addition, these results were further confirmed by immunohistochemistry (Figure 4F and 4G). These data indicate that CBX3 promotes colon cancer progression correlation with CDK6 and p21 expression.

### Decreased CDK6 expression promotes cell proliferation in the absence of CBX3

**Figure 5:**
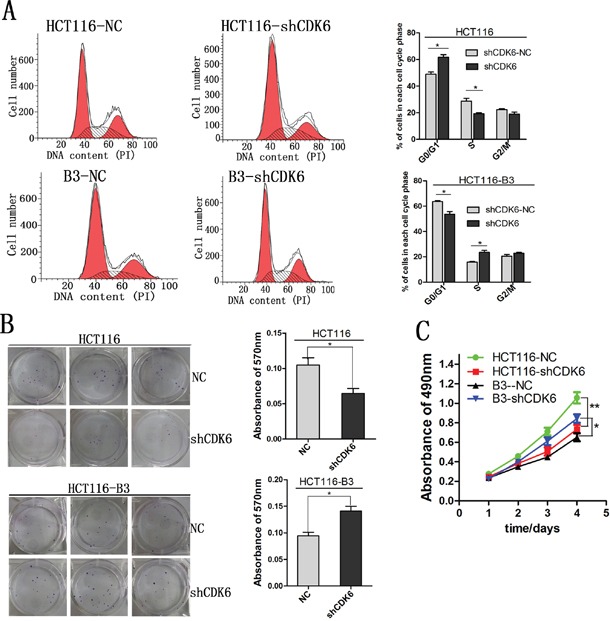
Decreased CDK6 expression promoted cell proliferation in the absence of CBX3 **A**. Cell cycle was analyzed by flow cytometry from CDK6 knocked down in HCT116 and HCT116-B3. Values were shown at different stages of cell cycle representing mean±SD from three independent experiments. *P<0.05 compared with control. **B**. Colony formation assay of HCT116 and HCT116-B3 with CDK6 knocked down or scrambled control cells. *P<0.05. **C**. CDK6 knocked down or scrambled control cell proliferation were detected by MTS. Absorbance of 490 nm were shown at different time points represented as mean±SD from three independent experiments. **P<0.01 compared with control.

Although CDK6 and p21 are important regulators of the G1-S cell cycle transition, they play opposing roles in cell cycle regulation. The study conducted by Kollmann et al. reveals an unexpected role for CDK6, as a transcriptional regulator on the p16ink4a promoter in BCR-ABL transformed B-cell leukemia/lymphoma cells. To investigate the specific biological role of CDK6 in colon cancer, we knocked down CDK6 expression in HCT116 and HCT116-B3 cells. We found that cell cycle G1-S phase transition was promoted in the absence of CBX3, but cell cycle was curbed in G1 phase in HCT116 (Figure 5A). In addition, cell proliferation was analyzed by MTS assay and colony formation. These results shown that cell growth in HCT116-B3-shCDK6 is faster than in control, but cell growth in HCT116-shCDK6 is slower than in control (Figure 5B and 5C). Taken together, our data indicates that CDK6 knocked down promotes colon cancer cell proliferation in the absence of CBX3.

### CDK6 regulates the expression of p21 by the kinase-independent function

**Figure 6:**
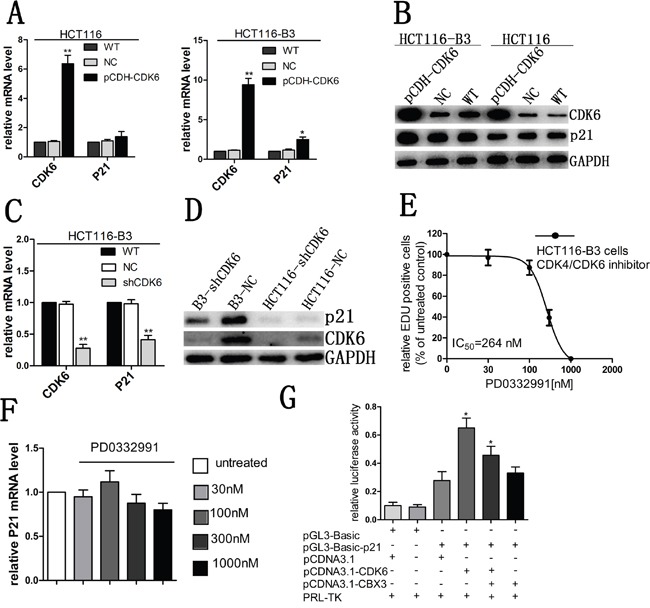
CDK6 regulated p21 expression in the absence of CBX3 **A**. qRT-PCR analysed CDK6 and p21 mRNA abundances in HCT116 and HCT116-B3 with CDK6 overexpression, the mean±SD from three independent experiments were shown. **P<0.01 compared with control. **B**. Immunoblot for CDK6 and p21 of HCT116 and HCT116-B3 with CDK6 overexpression. **C**. CDK6 and p21 mRNA level of HCT116-B3 cells with CDK6 knocked down were analyzed with qRT-PCR, the mean±SD from three independent experiments are shown.**P<0.01 compared with control. **D**. Immunoblot for CDK6 and p21 of HCT116-shCDK6, B3-shCDK6 and control cells. **E**. Dose-response curve of HCT116-B3 cells treated for 24 hr with the CDK6/4 inhibitor PD0332991 (n = 3). **F**. p21 mRNA levels of HCT116-B3 cells treated for 24 hr with 0, 30, 100, 300, and 1000 nM PD0332991 were analyzed by qPCR. P>0.05 compared with control. **G**. Luciferase reporter assays were performed in HCT116-B3 cells, co-transfected with pGL3-basic-p21 and pCDNA3.1-CDK6 or pCDNA3.1-CBX3, Empty pGL-basic and PCDNA3.1 plasmid was used as a negative control. *P<0.05.

We had previously found that CBX3 can inhibit the expression of CDK6 and P21 in colon cancer. To investigate the underlying mechanism, we examined p21 mRNA and protein abundances after increasing CDK6 expression. We found that enhanced CDK6 expression was consistently accompanied by elevated tumor suppressor p21 in the absence of CBX3 (Figure 6A and 6B) and the expression level of p21 was significantly reduced when CDK6 was knocked down (Figure 6C and 6D), but we did not find any change in HCT116. Meanwhile, CDK6 induced the expression of VEGF had not been observed (Supplementary Figure 3D).

Due to CDK6 and p21 performing opposing functions in cell cycle progression, we speculated that CDK6 may not act as a kinase dependent function cooperating with p21 to affect cell proliferation, but it was the kinase-independent function. A small molecule inhibitor (PD0332991) had been identified to directly bind the ATP binding pocket domain of CDK4 and CDK6. When we treated cells in HCT116-B3 with CDK4/CDK6 inhibitor, we found a significant reduction of cell proliferation correlating with increasing inhibitor concentration (Figure 6E). However, inhibitor treatment did not affect the abundance of p21 mRNA (Figure 6F). The results suggest that CDK6 regulates the expression of p21 by the kinase-independent activity.

To study the molecular mechanism of CDK6 regulation of p21 expression, a luciferase reporter assay was performed to analyze whether CDK6 enhanced the p21 promoter activity. The reporter plasmid was constructed and respectively cotransfected with two protein expression plasmids, including pCDNA3.1-CDK6 and pCDNA3.1-CBX3. We found that CDK6 could significantly increase p21 promoter activity. However, enhanced CBX3 expression markedly reduced p21 promoter activity (Figure 6G). Our data indicate that CBX3 prevents transcriptional regulation of CDK6.

### Inverse protein expression trends of CDK6 and p21 in human colon cancer tissue

**Figure 7:**
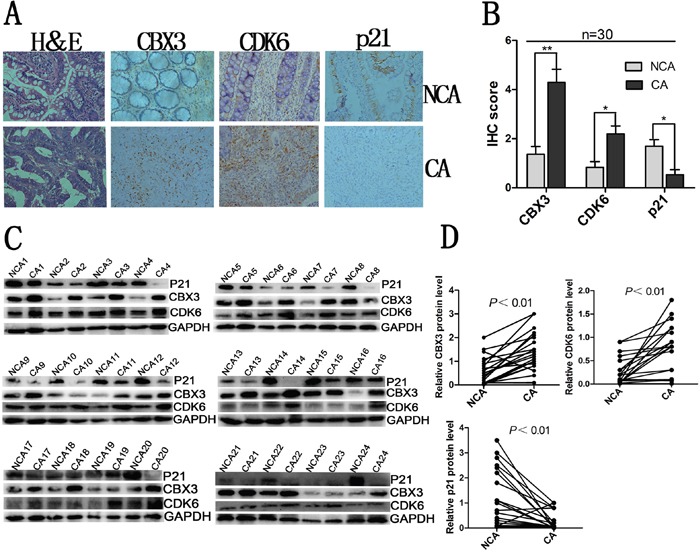
Inverse relationship between CDK6 and p21 expression in human colon cancer **A**. Hematoxylin and eosin (H&E) staining and immunohistochemical stainings of CBX3, CDK6 and p21 in colon cancer tissue and non-cancerous tissue. Representative micrographs including colon cancer tissue and non-cancerous tissue were shown, original magnification (×400). **B**. total IHC score of CBX3, CDK6 and p21 in colon cancer tissue and non-cancerous tissue (n=30), **P<0.01 or *P<0.05 compared with control. **C**. Immunoblot for CBX3, CDK6 and p21 of human colon cancer tissue (CA) and non-cancerous tissue (NCA) (n=24), GAPDH served as a loading control. **D**. CBX3, CDK6 and p21 protein expression levels by quantitation of density of protein bands from immunoblot in (C) in CA relative to NCA (n=24, **P<0.01).

To further confirm that CBX3 promotes colon cancer progression via regulating the expression of CDK6/p21, we examined the expression of CBX3, CDK6 and p21 in human non-cancerous tissue (NCA) and cancerous tissue (CA). We found high abundances of CDK6 accompanied by reduced or undetectable expression of p21, where CBX3 expression was significantly upregulated in colon cancer tissue (Figure 7A, 7B, 7C, 7D). However, we observed the opposite results in non-cancerous tissues. These results indicate that inverse protein expression levels of CDK6 and p21 may be regulated by CBX3 to disrupt a negative feedback loop in colon cancer.

## DISCUSSION

CBX3, from the heterochromatin protein 1 family, whose members are closely related to the biological processes in cells and have been proposed to be driving forces for some diseases when they are frequently expressed at aberrant levels. Although methylation of H3K9 is considered to be a characteristic of CBX3 elevation, the underlying mechanism for the preferential upregulation of CBX3 in carcinogenesis remains a mystery. In this study, we show that CBX3 protein levels are significantly increased in primary colon cancer tissues. Moreover, the higher expression of CBX3 is detected in poorly differentiated colorectal cancer tissues and the overall patient survival rate was significantly decreased [17]. These results suggest that CBX3 plays a distinctive role in the pathological process of cancer.

The deletion of CBX3 in colon cancer cells curb cell cycle progression (G1 phase to S phase) suppress cell proliferation *in vitro* and tumor growth *in vivo*, where CDK6 and p21 are consistently upregulated. On first thought, the result might appear paradoxical, because for a long time CDK6 was regarded as a mere homolog of CDK4 with overlapping functions in terms of Rb phosphorylation. The G1 kinases CDK4 and CDK6 are true cell-cycle kinases that regulate exit from the G1 phase of the cell cycle. They have received considerable attention as major drivers of cancer [21]. The study conducted by Kollmann et al. reveals several novel roles for CDK6 acting as the kinase-independent in transcription. Induction of p16 by CDK6 serves as a fail-safe mechanism that restricts excessive activity of CDK6 by forming a negative feedback loop [34]. In addition, CDK6 can bind to chromatin and recruit binding motifs of transcription factors such as c-Jun, AP-1, P65 to induce the expression of the genes VEGF-A, EGR1, IL. However, cell cycle-independent functions for CDK6 are not shared with CDK4 [21]. In our study, we found CDK6 regulates the expression of p21 by promoting its promoter activity when CBX3 is deleted and its expression is not affected by CDK6 kinase activity. Therefore, our finding can be rationalized by postulating a feedback loop including the upregulation of the tumor suppressor and cell-cycle inhibitor p21.

CBX3 is an evolutionarily conserved chromosomal protein that binds methylated H3K9, where it recruits cofactors or directly interacts with targets [17]. CBX3 is also a major component of heterochromatin and maintains the transcriptionally repressive heterochromatin structure to regulate gene transcription [3], including transcription silencing and retroviral silencing [30–32]. CBX3 has recently been proposed to have a close relationship with the tumor, such as non-small cell lung cancer, prostate cancer and breast cancer among others. However, the underlying mechanism of CBX3 to promote tumorigenesis is still largely unknown. In pancreatic cancer, NFATc2 binding to p15 promoter recruits histone methyltransferase Suv39H1 resulting in H3K9 trimethylation, which allows docking of CBX3 to the repressor complex giving rise to p15 silencing [33]. In the absence of CBX3, enforced CDK6 levels inhibit cell growth by upregulating inhibitor p21, but we can't do it in wild type cells, and promoter activity is suppressed by CBX3. In addition, we found that histologic sections of human colon malignancies have an inverse correlation of CDK6 and p21 expression, but CBX3 is in high abundance. Our data indicate that CBX3 participates in the silencing of p21 and prevents CDK6 transcriptional regulation.

**Figure 8:**
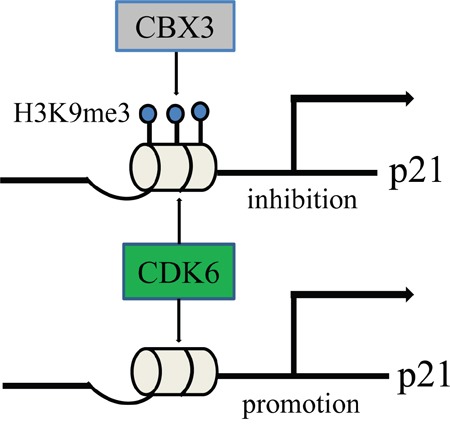
A hypothetical schematic sketch of CBX3/CDK6/p21 controlling cell cycle and proliferation

Cell growth is strictly regulated by cell cycle progression. CDK4/6–cyclinD complexes are controlled by two classes of inhibitors. CDK4 and CDK6 single knockout mice are viable. For one thing partially overlapping and redundant physiologic roles for CDK4 and CDK6, for another CDK6 may have a unique role to cell in a state, this is particularly evident in stem cells, where CDK6 is not required under homeostatic conditions but becomes critical under conditions of stress, such as oncogenic stress [35, 36]. CDK6 function as a transcriptional regulator linking cell-cycle progression to differentiation and other cellular functions is starting to be understood. The study of Kollmann et al. shows that CDK6 is capable of two opposing functions: it is able to inhibit or accelerate cell proliferation depending on whether p16 is present or not, but it is unclear what causes the initial upregulation of CDK6 and the silencing of p16. We find that p21 is nearly absent from colon cancer tissues, but CBX3 significantly increases. Promoter activity of p21 also significantly decreases with enhanced CBX3 expression. In addition, the histone marks H3K9me2/3 on the p21 promoter were also significantly reduced when CBX3 was downregulated [17]. We speculate that methylated H3K9 binding CBX3 in the p21 promoter prevents the function of CDK6 as a transcriptional regulator, in which it silences the expression of p21 and disrupts a negative feedback loop (Figure 8).

In conclusion, these findings provide a new perspective for understanding CBX3 as an oncogenic molecule in colon cancer. We have identified a novel role for CDK6 as a transcriptional regulator to link cell-cycle progression in colon cancer. Although we have demonstrated that CDK6 and p21 are regarded as the target of CBX3 regulation, it remains to be identified whether other inhibitors also are related to the CBX3 regulated network.

## MATERIALS AND METHODS

### Materials and reagents

Colon cancer cells were obtained from the tumor laboratory of the First Affiliated Hospital of Chongqing Medical University, China. The plasmid pX330-U6-Chimeric_BB-CBh-hSpCas9 was a gift from Feng Zhang (Addgene plasmid # 42230). Lentivirus plasmid pLKO.1 puro was a gift from Bob Weinberg (Addgene plasmid # 8453). Rabbit anti-CBX3 (A2248) was purchased from ABclonal Biotech Co (ABclonal, USA). The following antibodies were purchased from Abcam (Cambridge, MA): rabbit anti-CDK6 (ab124821), rabbit anti-p21 (ab109520). Rabbit anti-GAPDH (10494-1-AP) and rabbit anti-β-tubulin (10094-1-AP) were purchased from Proteintech Group, Inc. (Proteintech, China). Colon cancer tissue samples and matched adjacent normal tissues were derived from patients undergoing surgical procedures at the First Affiliated Hospital of Chongqing Medical University, China. All patients in the study provided written consent, and were approved by the Ethics Committee from the First Affiliated Hospital of Chongqing Medical University. Nude mice ages 4 to 5 weeks were purchased from Animal Experimental Center of Chongqing medical university. All animal care and handling procedures were handled in accordance with study protocols approved by the Ethics Committee of Chongqing Medical University.

### Construction of CRISPR/Cas9 vector

The CBX3 genome sequence was found in NCBI web-based database. The sgRNA was designed using the SAM Cas9 activator design tool (http://sam.genome-engineering.org/database/). Two sgRNA oligos were synthesized by Invitrogen. We followed the SAM target sgRNA cloning protocol to ligate the guide target sequence into pX330-U6-Chimeric_BB-CBh-hSpCas9 vector [27]. Px330 plasmids are sequenced using primers: F: AGGGATGGTTGGTTGGTGGG, R: CCAATCCTCCCCCTTGCTGT.

### Cell culture, transfections, cell clone, and infection

HCT116 cell lines were purchased from ATCC and cultured in DMEN (Gibco, USA) with 10% fetal bovine serum (BI, ISR) and incubated at 37°C in a humidified atmosphere containing 5% carbon dioxide. Each of the plasmids was transfected into the cell line with Lipofectamine 2000 (Invitrogen) according to the manufacturer's instructions. Px330 plasmid was transfected into HCT116. After 48 hours, the cells were cultured in a low density by trypsin digestion cell and add the puromycin to screen cells. Clone cell lines were picked using 10ul pipette tips into 96 well plates where cells are continuously cultured and gradually expanded. We obtained one cell line which we numbered HCT116-B3. Human CDK6 editing sequences were amplified by PCR with the following primers (see Supplemental Material, Table 1), which were cloned into the lentiviral vector plasmid pCDH-CMV-MCS-EF1-copGFP at unique *EcoRI* and *Bam*HI sites. A shRNA for human CDK6 targeting site was: 5′- GAGTAGTGCATCGCGATCTAA -3′ and was inserted into the *Spe*I/*Bam*HI sites in the pLKO.1 puro lentiviral vector [28]. Lentiviral particles were produced in HEK293T cells and infected HCT116 and HCT116-B3 cells.

**Table 1:** Amplify CDK6 primer and real-time PCR primer

CDK6	F: GGAATTC ATGGAGAAGGACGGCCTGTGCC (ECOR1) R: CGGGATCC TCAGGCTGTATTCAGCTC (Bamh1)		
name	primer	name	primer
CBX3	F:TGGCCTCCAACAAAACTACA R:TCCCATTCACTACACGTCGA	CDK6	F:CCAGGCAGGCTTTTCATTCA R:AGGTCCTGGAAGTATGGGTG
CCND1	F: ACAGATCATCCGCAAACACG R:GGCGGTAGTAGGACAGGAAG	CDH3	F: GTATGAGCTCTTTGGCCACG R: ATGGCATCATCCTCATCCGT
CCNE1	F:GGAAGAGGAAGGCAAACGTG R: TTTGTCAGGTGTGGGGATCA	CDC25A	F: CTACTGATGGCAAGCGTGTC R: TCTCTCTCACATACCGGCAC
P21	F: TGTCTTGTACCCTTGTGCCT R: GGCGTTTGGAGTGGTAGAAA	E2F1	F:CTTCGTAGCATTGCAGACCC R:AAAACATCGATCGGGCCTTG
CDK2	F: TCCGGATCTTTCGGACTCTG R: ACAAGCTCCGTCCATCTTCA	E2F3	F: ATCCCTAAACCCGCTTCCAA R:GAGGCCAGAGGAGAAAGGTT
CDK4	F: CTTCCCATCAGCACAGTTCG R: GGGGTGCCTTGTCCAGATAT	P16	F: CGATGTCGCACGGTACCT R: GACCTTCCGCGGCATCTAT

### Protein extraction and immunoblotting

All of protein extraction use RIPA cell lysis buffer (p0013B) from Beyotime Biotechnology according to the manufacturer's instructions. Protein samples (30-50 μg) were separated by SDS-PAGE, transferred onto PVDF membranes, and blocked with 5% milk. Primary antibodies were diluted with 5% BSA and 4°C overnight. Following incubation, the appropriate secondary antibodies were used from Proteintech (SA00001-2). Finally, chemiluminescence was visualized with Western Bright ECL HRP substrate (Advansta, USA). Equal loading of protein was verified by immunoblotting with anti-β-tubulin or anti-GAPDH antibody.

### RNA extraction, cDNA synthesis, and real-time PCR

Total RNA was isolated with Trizol reagent (Invitrogen, USA) according to the manufacturer's instructions. cDNA was synthesized from isolated RNA using PrimeScript RT Reagent Kit with gDNA Eraser (TAKARA, Japan) following the manufacturer's instructions. Real-time PCR was performed with UtraSYBR Mixture (CWBIO, China), and the relative levels of gene expression were determined by comparison with β-actin expression. The primer sequences used for qRT-PCR are listed in Table 1.

### Cell cycle analysis, MTS and EDU cell proliferation assay, colony formation assay

Cell cycle analysis was performed by flow cytometry from Academy of Life Sciences (Chongqing Medical University, China). Cell proliferation was quantified by CellTiter 96® Aqueous One Solution Cell Proliferation Assay (Promega, USA) and incorporation of 5-ethynyl-20-deoxyuridine (EdU) using an EdU Cell Proliferation Assay Kit (Ribobio, China) according to the manufacturer's instructions, respectively. Colony formation was observed with a crystal violet cell colony staining kit and cell growth was quantified by detecting absorbance value at 570 nm (GenMed, USA).

### Tumor xenografts in nude mice

3×10^6^ cells were diluted in 100 ul PBS, and each mouse was injected subcutaneously on the left side of the neck. Tumor growth rate was monitored by measuring tumor diameter (length=L, width=W) every 4 days. The tumor volume was calculated using the relationship: 1/2LW^2^. Mice were handled after 24 days, and tumors were collected following weight analysis.

### Immunohistochemical staining

All tissues were fixed with 10% paraformaldehyde, and embedded in paraffin wax. Paraffin sections were placed in incubators kept at 55°C for 4 hours. The sections were immersed in two consecutive washings in xylol for 20 min to remove paraffin. Sections were then hydrated with different concentrations of ethanol including 100%, 95%, 85%, 70% and deionized water respectively. The sections were immersed in citrate buffer solution (0.01 mol/L, pH 6.0) and heated to repair antigen, then 0.5% Triton-x-100 was incubated 30 min after washing in PBS. Biotin-streptavidin HRP detection systems (ZSGB, China) were then used to stain the section according to the manufacturer's instructions. In parallel, tissue samples in which the primary antibody was replaced by PBS served as negative control.

### Immunofluorescence

Cells were incubated on the glass coverslip. Cells were fixed with fresh 4% paraformaldehyde, permeabilized and blocked with 0.5% Triton-X-100 and 4% BSA, respectively. Primary antibody and secondary antibody were diluted with 4% BSA. DNA was stained with 4′,6-diamidino -2-phenylindole (DAPI) (Beyotime, China). A fluorescence microscope was used to detect the targeted protein.

### Luciferase assay

The pGL3-Basic, pCDNA3.1and pRL-TK plasmids were purchased from Invitrogen (USA). The p21 promoter sequence was identified in the NCBI web database and the sequence (−1200-+300) was amplified using the following primers: FCGGCTAGCGACAATGCTTAGTTCAGATAC, R: CC CAAGCTTTACCCAGACACACTCTAAGG. HCT116 cells were co-transfected with pGL3-basic-P21 and pCDNA3.1-CDK6 or pCDNA3.1-CBX3. Each sample was also co-transfected with pRL-TK. Cells were harvested 48 hr later and assayed with the Dual Luciferase Reporter Assay System (Promega, Madison, WI) according to the manufacturer's instructions. Relative luciferase activity was normalized to renilla luciferase activity. The assay was repeated three times in independent experiments.

### Statistical analysis

All statistical analysis was performed using SPSS16.0 (SPSS Inc. Chicago, IL, USA). Data were presented as mean ± SD differences between multiple means were evaluated by two-tailed Student's t-test. A value of P<0.05 was considered statistically significant. IHC-score was performed as described previously [29]. Student's t-test was performed for IHC-score and a value of P<0.05 was considered statistically significant.

## SUPPLEMENTARY MATERIALS FIGURES


