# Supplementary material for: CBX3 promotes colon cancer cell proliferation by CDK6 kinase-independent function during cell cycle
Source: Oncotarget. 2017 Feb 10;8(12):19934–46. doi: 10.18632/oncotarget.15253 (PMC5386735; doi:10.18632/oncotarget.15253)
Supplement: Supplementary file 1 [file oncotarget-08-19934-s001.pdf]

CBX3 promotes colon cancer cell proliferation by CDK6 kinase-independent function during cell cycle

SUPPLEMENTARY FIGURES

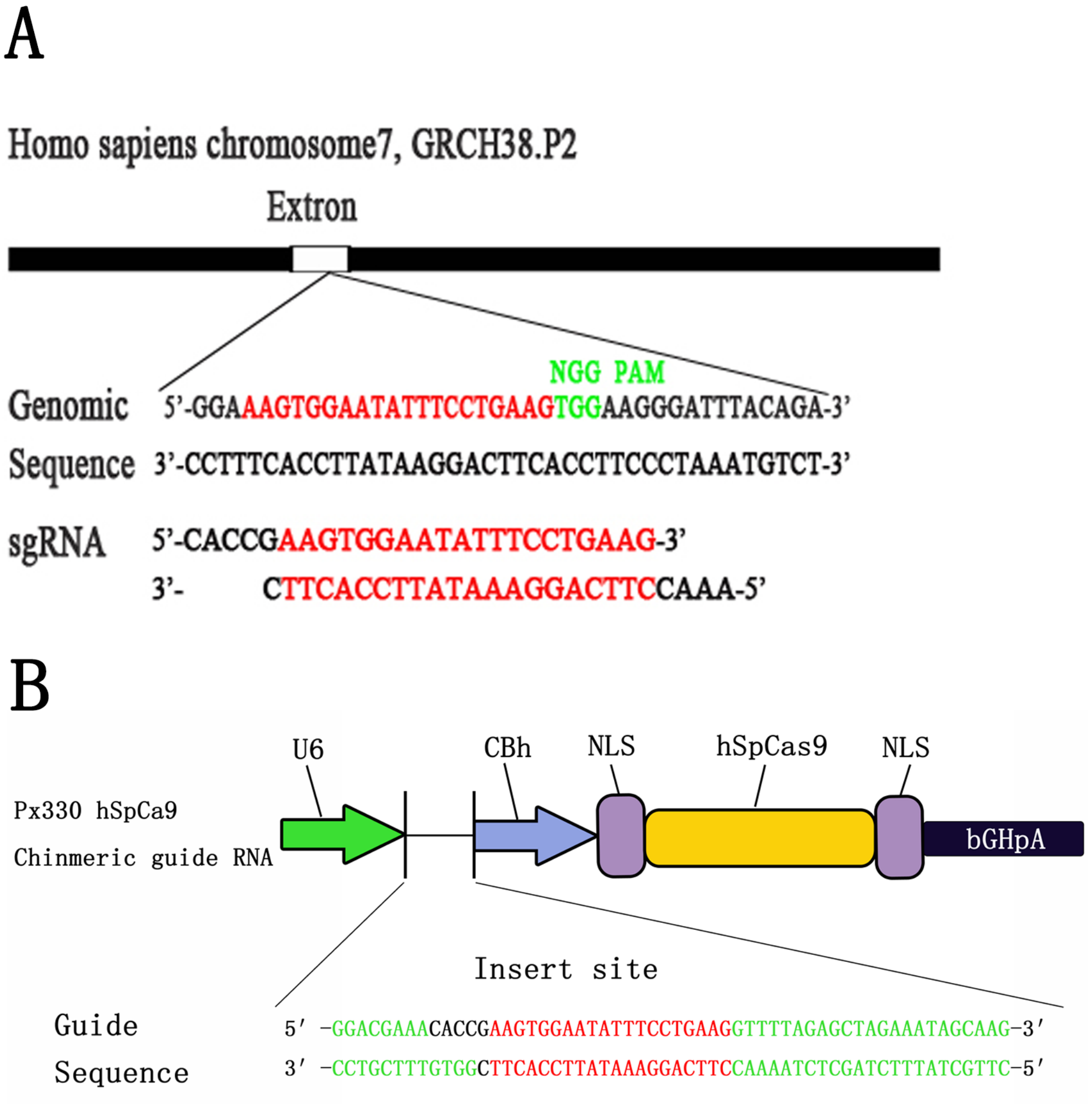

Supplementary Figure 1: sgRNA was designed **A.** and Px330 plasmid was constructed **B.**

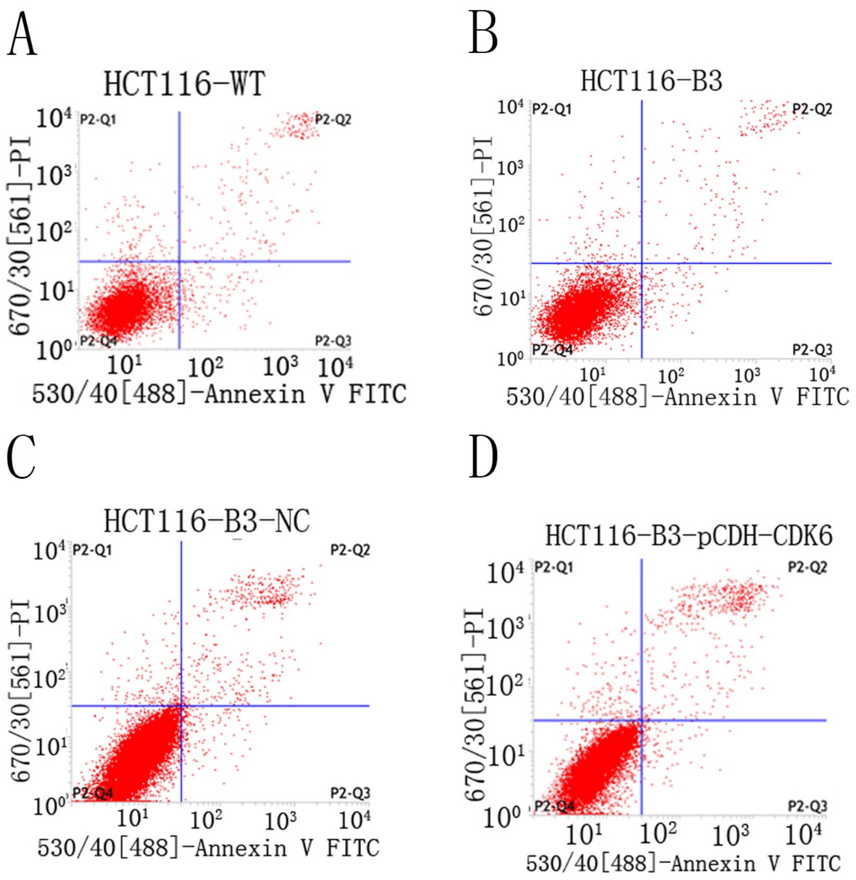

Supplementary Figure 2: Effect of CBX3 **A, B.** and CDK6 **C, D.** on HCT116 and HCT116-B3 cells apoptosis.

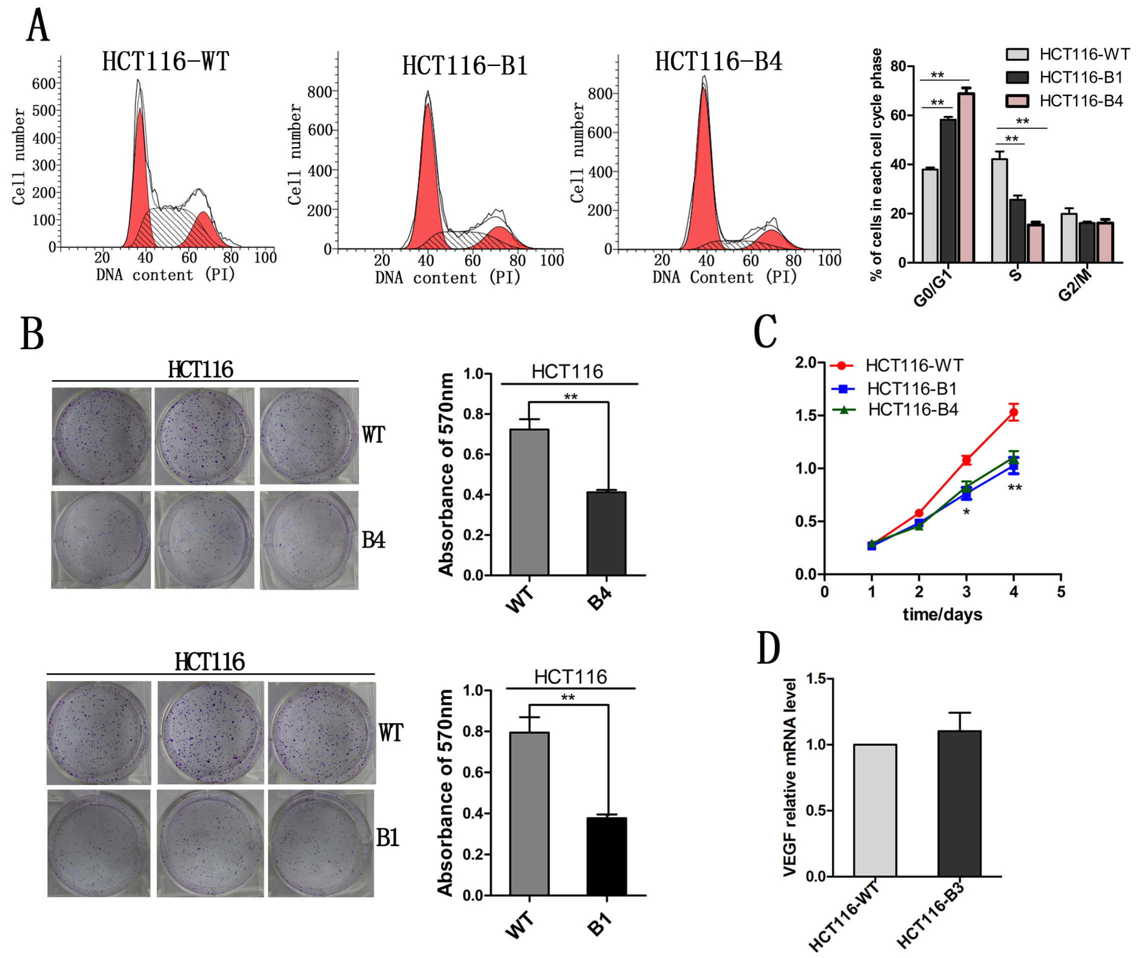

Supplementary Figure 3: CBX3 deletion inhibited cell cycle progression and proliferation *in vitro*. A. Flow cytometry assays were performed to analyze cell cycle in HCT116-WT and B1 and B4 cells. Values at different stages of cell cycle represent mean±SD from three independent experiments. **P<0.01 compared with control. **B.** Colony formation assays were performed using HCT116-WT, B1 and B4 cells. Colony number were analyzed by measuring absorbance at 570 nm The results were shown as mean±SD from three independent experiments. *P<0.05 compared with control. **C.** Colon cancer cell proliferation were detected by MTS and absorbance at 490 nm at different time points is shown. Each data point represents mean±SD from three independent experiments. **P<0.01 compared with control. **D.** qRT-PCR analysis mRNA level of VEGF from HCT116-WT and B3 cell lines. The mean±SD were shown from three independent experiments. P>0.05 compared with the control.
